# Morphometry and Molecular Identification of *Haemonchus* Cobb, 1898 (Trichostrongylidae: Nematoda) Isolates from Small Ruminants in Tanzania Based on Mitochondrial *cox* 1 and rRNA-ITS genes

**DOI:** 10.1155/2023/1923804

**Published:** 2023-01-16

**Authors:** Barakaeli Abdieli Ndosi, Dongmin Lee, Mohammed Mebarek Bia, Heejae Yang, Min-Ji Hong, Sungbo Seo, Hansol Park, Keeseon S. Eom

**Affiliations:** ^1^Department of Parasitology, Parasitology Research Center and International Parasite Resource Bank, Chungbuk National University, School of Medicine, Cheongju 28644, Republic of Korea; ^2^Tanzania Wildlife Management Authority, P.O. Box 2658 Morogoro, Tanzania; ^3^Cocoon Inc. #704, 194-41, Osongsaengmyeong 1-ro, Osong-eup, Heungdeok-gu, Cheongju-si, Chungcheongbuk-do, Republic of Korea

## Abstract

The genus *Haemonchus* is the major abomasal parasite of ruminants responsible for substantial economic losses in tropical and temperate regions. This study was conducted to clarify the morphometry and molecular characterisation of *Haemonchus* species isolated from sheep in Babati district, Tanzania. A total of 486 trichostrongylid nematodes were recovered from five sheep. Of the total worms, 106 nematodes were distinguished by 37 males and 69 females. The asymmetrical length of dorsal ray and the distance of bulb at the apex of spicules were used for identification of males. In females, the linguiform vulvar flap was the most predominant with 33 out of 69 (48%) compared with knobbed morph type which was 25/69 (36%) and smooth morph type with 11/69 (16%). Partial *cox*1 sequence fragments of *Haemonchus contortus* isolates showed 98.8%, 99.3%, 99.7%, 99.5%, 99.3%, and 98.4% in male, smooth, knobbed, linguiform A, linguiform B, and linguiform C, respectively; with the average nucleotide divergence ranged from 1.03 to 2.35%. The amplified fragments of ITS-2 genes in knobbed, linguiform A, and smooth morphotypes revealed 99.4%, 98.5%, and 98.3%, respectively. Phylogenetic analysis was evaluated by employing Bayesian inference and maximum-likelihood, and the tree was distinctly separated into three clusters focusing on *H. contortus* in cluster I within the family Haemonchidae. Genetic drifting, mutation, and modification of the morphological features of the *Haemonchus* species described to have an impact on the development of drug resistance. Species identification is necessary to understand which species infect animal host. We recommend more studies on the parasites intensity and the strategies for controlling *Haemonchus* species in Tanzania.

## 1. Introduction

The genus *Haemonchus* (Trichostrongylidae) has bloodsucking nematodes of domestic and wild ruminants. The adult worm is cylindrical in shape and yellowish in colour with alternating red and white barber-pole; Male spicules have bulb shaped blunt pointed anterior end [1]. The *Haemonchus* species are potential harmful and significant threat in tropical, subtropical, and warm temperate regions [2]. The genus is orally transmitted to all classes of ruminants by consuming contaminated pastures with L3 infective larvae that develop into adult worm whilst burrow into the internal layer of the abomasum [3]. The adult worm feeds on the host's blood in the abomasum causing anaemia and oedema, which reduce animal production and ultimate deaths [4].

The identification of *Haemonchus* species has controversial ideal in their morphology and host specificity. However, *Haemonchus contortus* (Rudolphi, 1802) Cobb, 1898 has been reported in sheep (*Ovis aries*) and wild ruminants [5, 6]; *Haemonchus longistipes* Railliet & Henry, 1909 in camel (*Camelus dromedarius*) [7, 8]; and *Haemonchus placei* (Place, 1893) Ransom, 1911 in white-tail deer (*Odocoileus virginianus*), Pronghorn antelope (*Antilocapra americana*), *O. aries*, and domestic cattle (*Bos taurus*) [9]. *Haemonchus mitchelli* LeRoux, 1929; *Haemonchus okapiae* Van den Berghe, 1937; and *Haemonchus similis* Travassos, 1914 have been reported in *O*. *aries* and *B*. *taurus* in Brazil, Central and South America, Asia, Atlantic, and Pacific islands [10, 11].

Synlophe patterns, spicules morphometry, vulvar flap, and cervical papillae are important morphological features in the identification of *Haemonchus* species [12]. In addition, the host feeding habit [13], host immunity [14], host strain [15], host gender [16], and coexisting parasitic infection described to manipulate their morphological features [17]. The clarification of *Haemonchus* species by morphological features is still debatable, which demand the use of molecular techniques to unveil specific and intraspecific variations associated with their geographical distribution [18]. The investigation on nematodes through nucleus and mitochondrial deoxyribonucleic acid (DNA) provides genetic information on the infection of subpopulation of a parasitic species to the host species [19].

*H. contortus*, *H*. *placei*, and *H*. *similis* have been reported in domesticated ruminants [20], with limited clarification on their morphological features in Tanzania. Regardless of developing low-cost molecular tools for investigating *Haemonchus* species with other trichostrongylid nematodes [21], there is no clear description on the morphology and phylogenetic information of *Haemonchus* species isolated from sheep and other small ruminants in Tanzania.

Therefore, this study was taken to clarify the identification of *Haemonchus* species based on morphology and genetics by using mitochondrial cytochrome oxidase subunit 1 gene (*cox* 1) and the ribosomal RNA-internal transcribed spacer (rRNA-ITS).

## 2. Materials and Methods

### 2.1. Study Area and Animal Selection

This study was conducted in three villages namely Kiru, Ayasanda, and Magugu villages in Babati district located in Manyara region. Babati district is confined between 3° and 4° South and 35° and 36° East [22]. Five sheep were selected randomly from the selected villages from June 2020 to January 2021. The selected animals were 8–10 months old considering the choice of young animals and those that have not been recently dewormed [23].

### 2.2. Climate Condition of the Area

Warm-summer Mediterranean and tropical climate is mostly dominated in the area. The warm season lasts for 5.5 months, from October to March, with an average daily temperature above 27°C [22]. The cool season lasts from June to August, with an average daily temperature below 23°C. The rainy period of the year lasts for 7.3 months, from October to May, whereby the high rainfall period begins from March and ends in May. The dry period lasts for 4.7 months, from May to October [24].

### 2.3. Animal Processing

The isolation of worms was conducted based on Hansen and Perry [23] and Maff [25] with some modification. The gastrointestinal tracts of selected sheep were double ligated at the abattoir into three sections to prevent mixing of the abomasal, small intestinal, and large intestinal contents. Each tract was placed in a bucket, labelled before being processed. The nematode worms were isolated, counted, and placed in collecting tubes containing 70% ethanol for molecular studies and 10% formalin for morphological studies [23].

### 2.4. Morphological Identification of *Haemonchus* Species

Prior to identification, the nematode worms were washed in normal saline to free them from mucus and then fixed in hot 70% ethanol. Dorsal ray and spicules were considered as important key for identification for the male worms. The posterior parts of the male worms were cut before the bursa and mounted in lactophenol for clear examination of the spicules under a microscope [26]. For the female worms, the cuticular process and vulvar shapes were examined based on Rose [27] and Le Jambre and Whitlock [28] procedures.

### 2.5. Molecular Identification of *Haemonchus* Species

#### 2.5.1. PCR and DNA Sequencing

Collected nematodes were sorted by grouping parasites per host species. Individual nematode was selected from each host group and washed in Phosphate buffered saline (PBS). Prior to the extraction, worms were ground with a pestle in adaptive transport layer buffer and Proteinase K in 1.5 ml microcentrifuge tube and allowed to melt into suspension overnight at 56°C. The incubated nematode sample was extracted using QIAamp DNA mini-Kit following the manufacturer's procedures (Qiagen, Valencia, CA, USA). Genomic DNA was dissolved in 50 *μ*l of Tris-EDTA (TE) buffer (10 mM Tris/1 mM Ethylenediaminetetraacetic Acid (EDTA)). The mitochondrial *cox* 1 fragment was amplified by primers JB3 (5′-TTT TTT GGG CAT CCT GAG GTT TAT-3′) and JB4.5 (5′-TAA AGA AAG AAC ATA ATG AAA ATG-3′) [29]. The ITS gene primers NC2 (TTA GTT TCT TTT CCT CCG CT) and NC5 (GTA GGT GAA CCT GCG GAA GGA TCA TT) were used for amplification of rRNA-ITS genes [30]. The polymerase chain reaction (PCR) amplification was performed using 50 ng of genomic DNA template in 25 *μ*l reaction mixtures consisting of 1 *μ*l of each primer (10 pmol), 1 *μ*l of generic DNA, 2.5 *μ*l of 10× buffer [200 mM Tris–HCl (pH 8.4) and 500 mM KCl], 12.25 *μ*l of 2× buffer (MgCl_2_, dNTP) and 1.25 units *Taq* polymerase (Takara Bio Inc.^®^, Kusatsu, Shiga, Japan), and 6 *μ*l of distilled water.

The genomic DNA was initially denaturized at 95°C for 2 minutes followed by 30 cycles of denaturation at 95°C for 60 seconds, annealing at 55°C for 60 seconds, and extension at 72°C for 60 seconds with a final extension at 72°C for 7 minutes. PCR condition for ITS-2 region was 3 minutes at 95°C, over 35 cycles of 1 minute at 95°C, 1 minute at 58°C, 60 seconds at 72°C, and a final extension step of 10 minutes at 72°C.

#### 2.5.2. DNA Sequence Analyses

DNA sequences of the mitochondrial *cox*1 and ITS-2 were assembled using Geneious R9.1 (Biometer, Auckland, New Zealand). These sequences were compared with the published *cox* 1/ITS gene sequences from the GenBank: *H*. *contortus* (EU346694/AB908961), *H*. *placei* (NC029736/JN128896), and *Teladorsagia circumcincta* (CB036905); Trichostrongylidae, *Trichostrongylus axei* (MW051254/KC337056), and *Trichostrongylus vitrinus* (MW051255/JF680986); and the outgroup of trematodes the *Paragonimus westermani* (NC O27673/KT020830) and *Clonorchis sinensis* (JF729304/AF040935).

Phylogenetic analysis was evaluated by employing Bayesian inference (BI) and maximum-likelihood (ML) using the partial sequences of *cox* 1/ITS 1 in the Molecular Evolution Genetics Analysis (MEGA) software version 7.0 [31]. The HKY+G substitution model was used for sampling of *cox*1 and ITS sequences. BI analyses were used in the Bayesian Evolutionary Analysis Sampling Trees program version 1.10.4 [32]. The HKY substitution model sampling was chosen according to the MEGA. The nodes were assessed by bootstrapping with 1,000 pseudoreplicates.

## 3. Results

**Figure 1:**
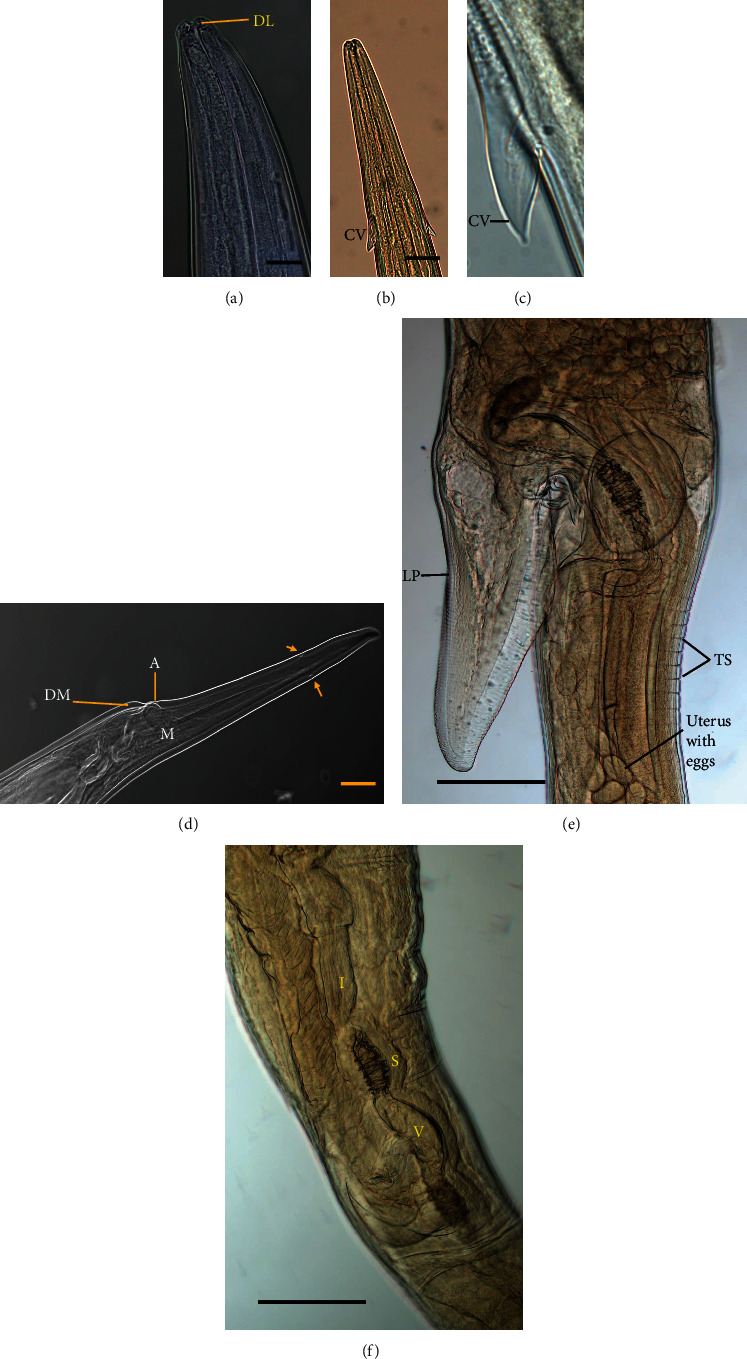
(a) Anterior end; dorsal lancet (DL). (b) and (c) Cervical papillae (CV). (d) Posterior end; anus (A); phasmids (arrow); dorsal rim cover the anus (DM); muscle (M; scale bar 50 *μ*m). (e) Linguiform process (LP); transverse cuticle striations (TS). (f) I: anterior infundibulum, S: anterior sphincter, and V: vestibular (scale bar 200 *μ*m).

A total of 486 trichostrongylid nematodes were recovered from five sheep in Babati district abattoir. Of the total worms recovered, 106 nematodes were distinguished by 37 males and 69 females. The high number of parasites recovered in abomasa were similar size except those from female sheep observed to be larger than in the male sheep. The faint pale-yellowish and slender nematodes tapering towards both ends with alternating red and white “barber-pole” in female were observed, whereas the filiform in the anterior parts of the males and bursal lobes in the posterior part were observed. The buccal cavity was relatively small without buccal capsules; rather there is pronounced dorsal lancet protracted from dorsal wall (Figure 1). Both male and female worms had cervical papillae with spine structure-like protruding outside more or less parallel to longitudinal cuticular ridges, with highly visible transverse striations on the body (Figure 1).

**Figure 2:**
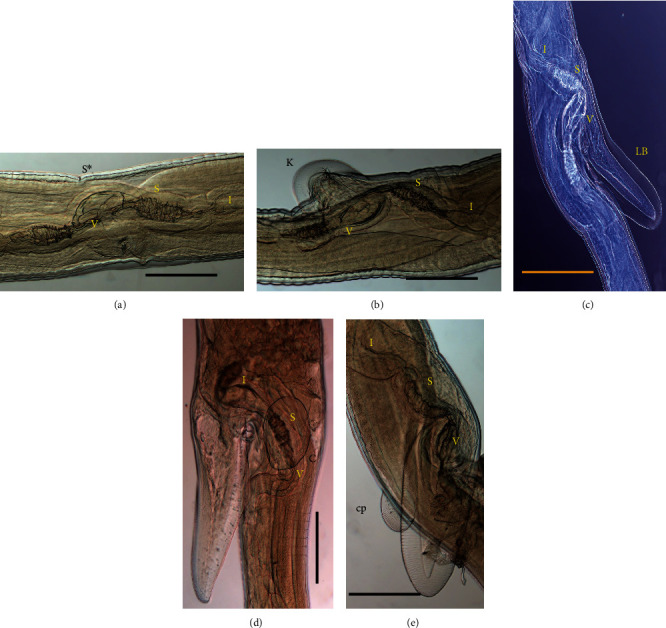
(a) Smooth (S∗). (b) Knobbed (K). (c) Linguiform B (LB); (d) Linguiform A with one cuticular inflation (C; scale bar 50 *μ*m). (e) Linguiform C with cuticular raised from the linguiform process (cp). I: anterior infundibulum; S: anterior sphincter; V: vestibular (scale bar 200 *μ*m).

The morphmetic features of female Haemonchus contortus associated with the vulvar position with or without prominent features along its structure. The linguiform vulvar flap was the most predominant count for 33 out of 69 (48%) compared with knobbed females with 25/69 (36%) and smoothed females that count for 11/69 (16%; Figure 2). In addition, the linguiform vulvar flap also could be categorized into three subtypes the linguiform A, B, and C (Figure 2). For the purpose of this study, the linguiform A is the vulvar morph type with one cuticular inflation; linguiform B is the vulvar flap with no cuticular inflation; and linguiform C is the vulvar flap with cuticular inflation raised from the linguiform process (Figure 2).

### 3.1. Female

Morphometric females ranged from 14,800 to 27,200 *μ*m, with an average of 18,500 *μ*m in length and 313–374 *μ*m in width. The cervical papillae measured 111–212 *μ*m, with a long club shaped oesophagus measuring 1,111–1,414 *μ*m in length and excretory pore measuring 131–313 *μ*m (Table 1). The vagina length is associated with linguiform, infundibulum length, sphincter length, and vestibular. The vulvar flap is well demarcated with or without linguiform processes (Figure 2; Table 2). The tail is long without projectory spine with simple dorsal and muscular ventral rims developed along the anal pore at the posterior end of the tail (Figure 1).

### 3.2. Male

**Figure 3:**
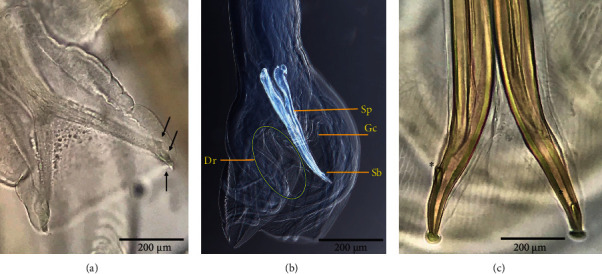
Dorsal ray of *H. contortus*. (a) Subdivision of branch-nodes parallel to the long-branched lobe to the margin (arrows). (b) Distal end of male dorsal ray (Dr); genital cone (GC) dorsally; spicules (Sp) spicule barbs (Sb). (c) Spicule showing spicule barbs variation at the apex (asterisk; scale bar 200 *μ*m).

The filiform males were small compared with females, with an average length of 14,039 *μ*m and width of 298 *μ*m. The cervical papillae are 210 *μ*m from the anterior end along with the oesophagus with a length of 1,232 *μ*m; in the posterior end, the male bursa revealed elongated lobes with long and muscular slender rays of variations in their lobes (Figure 3). The dorsal ray is asymmetrically branched to its length. Each branch divides again approximately at the end of its length giving rise to a short external branch node parallel to the long branches projecting to the dorsal lobe margin. The two spicules measure 434 *μ*m with small bulb and pore near its end supported by gubernaculum, which is spindle shaped in dorsal view narrowing to the posterior extremity measuring 212 *μ*m (Figure 3; Table 1).

### 3.3. Molecular Descriptions

**Figure 4:**
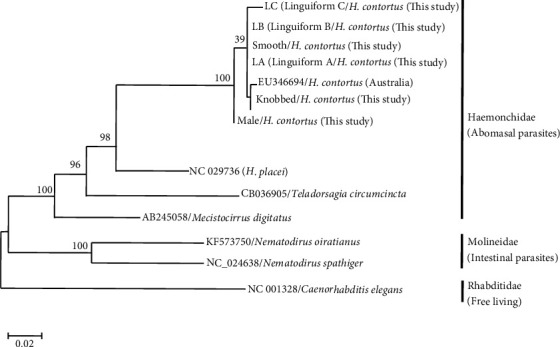
Inferred phylogenetic relationship of *Haeamonchus contortus* (present study) showing the relationship with *H. placei* with other abomasal nematodes of Haemonchidae in cluster I and intestinal parasites of Molineidae family in cluster II, and the Rhabditidae a free living as outgroup based on *Cox*1 by BI and ML analyses.

Phylogenetic analysis was determined by the ML and BI. The phylogenetic tree was distinctly separated into three clusters (Figure 4). Cluster I was abomasal nematodes, the Haemonchidae with *H*. *contortus* (EU346694), *H*. *placei* (NC029736), *T. circumcincta* (CB036905), and *Macrostomus digitatus* (AB245058); cluster II was intestinal parasites of the family molineidae, with *Nematodirus oiratianus* (KF573750) and *Nematodirus spathiger* (NC_024638); and the outgroup of the free nematode *Caenorhabditis elegans* (NC001328). Cluster I was subdivided into three clades: clade I with *M. digitatus*, clade II the *T. circumcincta*, and clade III the *H*. *placei* and *H*. *contortus*. The *H. contortus* was subdivided into groups of males, linguiform C, linguiform B, linguiform A, smooth and knobbed morph types in females (Figure 2). The average nucleotide divergence among the *H. contortus* individuals in the present study in cluster I is 1.03%, whereas with the entire population of *H. contortus* and *H. placei* retrieved from the GenBank is 10.2–11.2% (Table 3).

**Figure 5:**
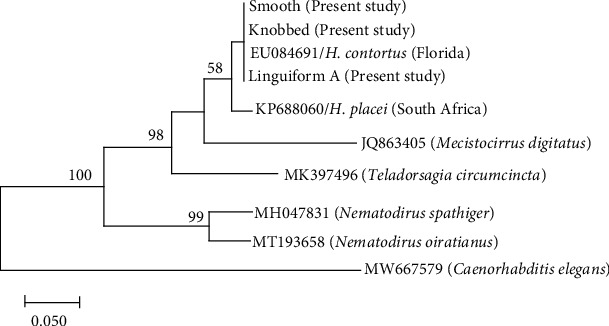
Phylogenetic relationship of *H*. *contortus* (present study) showing the relationship with *H. placei* with other abomasal nematodes (Haemonchidae) based on ITS-2 by BI and ML analyses.

The nucleotide identities between the *Haemonchus* species were revealed by percentage composition of 98.6–99.7%. Partial *cox* 1 sequences of 344, 277, 317, 365, 302, and 317 bp fragments of *H. contortus* isolates in the present study showed 98.8%, 99.3%, 99.7%, 99.5%, 99.3%, and 98.4% in male, smooth morph type, knobbed morph type, linguiform morph type A, linguiform morph type B, and linguiform morph type C, respectively, similar to *H. contortus* from Australian isolate (EU346694). The sequences were deposited in Genbank with accession numbers OK236357, OK178541, OK178542, OK178543, OK236356, and OK236358 for the male, knobbed morph type, linguiform A, linguiform B, smooth morph type, and linguiform C, respectively. The amplified 532, 520, and 522 bp fragments of ITS-2 genes in knobbed morph type, linguiform A, and smooth morph type *H. contortus* isolates were aligned with *H. contortus* from Florida (EU084691) revealing 99.1%, 98.8%, and 98.3%, respectively (Figure 5). The sequences were deposited in Genbank with accession numbers OK181226, OK181227, and OK181228 for knobbed, linguiform A, and smooth morph type, respectively.

## 4. Discussion

Morphological study has been widely used for understanding the biology, population, and ecological adaptation of *Haemonchus* species infestation in the small ruminants [28]. The main focus is to reduce the burden of worms in the gastrointestinal tract of small ruminants by rescinding morphological features, such as length and width of the worm, cervical papillae, vulvar flap, spicule, and gubernaculum length [36]. The asymmetrical length of dorsal ray with branch nodes towards the dorsal lobe margin and the distance of bulb at the apex of spicules were important structures for the identification of males. The comparative study of the female morph types, such as knobbed, smooth, and linguiform, is related to geographical locations and population density of the *Haemonchus* species [37, 38]. In addition, the presence of morph types with additional inflations in females is associated with the multiple phenotypic expression caused by adaptive characteristics of the *Haemonchus* species based on environmental factors [39]. We provide the phylogenetic relations among the linguiform and other morphometric types observed in this study. Linguiform was highly observed compared with other morph types possibly caused by the dominance modifiers that differ from locality [40]. It is still uncertain to conclude the significance of phenotypes as genetic markers due to limited research illustrating the dominance hierarchy of *Haemonchus* species in Tanzania.

The availability and use of genetic information are vital tool for the management of parasitic diseases for easy identification of specific parasite infecting the host [41]. The molecular information works as promising outstanding markers for species description and determination of genetic population. For instance, the use of ITS genes as one of the most variable nuclear loci for evolutionary studies becomes more important in discriminating nematode species [42, 43]. The use of *cox* 1 and ITS-2 with some portion of 5.8S sequences in this study provides a significant step in discriminating *H*. *contortus* especially in Tanzania.

The variability of *H. contortus* in the present study is associated with the area of study that the sheep were selected randomly in the population, which were not recently dewormed and shared the same pastures with other ruminants, and the timing of sampling, which is associated with the transmission risks (June to January) similar to what has been proposed in previous studies [19, 42]. In addition, the use of *cox* 1 and ITS-2 with some portion of 5.8s, which are stable conserved regions among the trichostrongyloid genomes, revealed the clear relationship among the monophyletic in Haemonchidae. The present study showed a high percentage of similarity with *Haemonchus* species that was confirmed from previous study to contribute to the development of haemonchosis. The present study is signifying that the comparative sequence analysis of *Haemonchus* species is a powerful tool for inferring the function *cox*1 and ITS-2 with portion of 5.8s as the novel functional genes for identifying *Haemonchus* species. During sampling, dewormed sheep were selected randomly in the same pastures with other ruminants and the timing of sampling was associated with the transmission risks (June to January) similar to what has been proposed in previous studies [44–46] on the comparison of Haemonchidae and Molineidae families to see the genetic relationship on the site of infection that *H*. *contortus* infect abomasum are closer to intestine parasites. The present study is emphasising on the use of *cox* 1 sequences due to its high affinity in the accumulation of substitutions more than ITS genes. However, the use of ITS genes has significant importance on the quick distinguishing between known species (lower level of intraspecific polymorphism) [19].

## 5. Conclusion

The continue persistence of *Haemonchus* is related to the genetic drifting, mutation, and modification of their morphological features described to have impact on the development of drug resistance. Species identification is necessary to understand which species infect animal host [46]. The use of molecular marker in identification could provide more information on the genetic variation for the species that could provide more information on the treatment procedures include drug discovery. Therefore, we recommend more studies on the parasites intensity and the strategies for controlling *Haemonchus* species in Tanzania.

## Figures and Tables

**Table 1:** Morphometric features of male and female *Haemonchus contortus* collected in Tanzania compared with other locations.

Morphological features	Sahai and Deo (1964) [33]		Soulsby (1982) [34]		Lichtenfels (1994) [35]		Current study	
(*μ*m)	Male	Female	Male	Female	Male	Female	Male	Female
Number examined	N/A	N/A	N/A	N/A	23	22	27	69
Body length	14,000–17,000	20,000–27,000	10,000–12,000	18,000–30,000	11,100–17,000	14,800–27,200	14,039	14,800–27,200
Width	199–265	215–332	—	—	—	—		313–374
Nerve ring	—	—	—	—	188–326 (254)	210–394 (271)	111	81–151
Excretory pore	—	—	—	—	232–356 (285)	205–400 (290)	151	131–313
Cervical papillae	—	—	—	—	271–462 (354)	243–484 (363)	210	111–212
Oesophagus length	1,444–1,743	1,162–1,662	—	—	1.09–1.55 (1.26)	243–484 (363)	1,232	1111–1,414
Anterior infundibulum length	N/A	—	N/A	—	N/A	188–394 (272)	N/A	101–313
Anterior sphincter length	N/A	—	N/A	—	N/A	154–284 (222)	N/A	141–232
Vestibular length	N/A	—	N/A	—	N/A	150–263 (198)	N/A	156–222
Vagina length	NA		N/A	—	N/A	75–188 (110)	N/A	80–202
Tail length	N/A	415–513	N/A	490–550	N/A	251–530 (396)	N/A	56–596
Gubernaculum	199–349	N/A	—	N/A	195–255 (221)	N/A	212	N/A
Spicule's length	398–448	N/A	460–506	N/A	383–475 (425)	N/A	434	N/A
Dorsal ray							181.8	N/A
Genital cone							50.5	N/A
Description	Sheep and goat		Sheep and goat		Several		Sheep	
Locality	India		London		Several		Tanzania	

N/A: not applicable; —: not indicated; Several: different hosts collected from various regions include United State of America, Brazil, Australia, South Africa, Sierra Leone, Puerto Rico, and Guyana.

**Table 2:** Morphometric of female *Haemonchus contortus* showing vulvar position and accompanied features of smooth, knobbed, and linguiform morphotypes.

Morphological features (*μ*m)	Smooth	Knobbed	Linguiform		
Total count	11 (16%)	25 (36%)	33 (48%)		
			LA	LB	LC
Body length	24,442	19,241	20,604	21,362	20,402
Width	354–374	354–364	343	313	354
Nerve ring	101–109	81–121	151	101	101
Excretory pore	212–253	202–273	171	313	131
Cervical papillae	111–253	111–182	212	202	202
Oesophagus length	1,111–1,313	1,212–1,131	1,232	1,212	1,414
Anterior infundibulum length	101–313	202–156	121	141	242
Anterior sphincter length	141–182	156–232	182	152	222
Vestibular length	162–172	156–182	222	202	202
Vagina length	80–91	152–202	131	152	202
Tail length	56–505	455–556	596	556	475
Description	No morph type along the vulvar flap	Protrude along the vulvar flap	Possess one cuticular inflation	No cuticular inflation	Cuticular inflation raised from the linguiform process

LC: linguiform vulvar morph type-C; LA: linguiform vulvar morph type-A; LB: linguiform vulvar morph type-B.

**Table 3:** Genetic divergence among the *Haemonchus contortus* male and female morph types isolated from Babati district, Tanzania.

	Male	LC	LA	LB	Smooth	Knobbed	EU346694 (*H. contortus*)	NC_029736 (*H. placei*)
Male		1.6	0.6	0.7	0.7	0.9	1.2	10.2
LC	1.6	1.6	0.9	0.7	0.4	1.3	1.6	11
LA	0.6	0.9		0	0	0.3	0.5	10.4
LB	0.7	0.4	0		0	0.7	0.7	10.9
Smooth	0.7	0.4	0	0		0.4	0.7	11.2
Knobbed	0.9	1.3	0.3	0.4	0.4		0.3	11
EU346694 (*H. contortus*)	1.2	1.6	0.5	0.7	0.7	0.3		10.2
NC_029736 (*H. placei*)	10.2	11	10.4	10.9	11.2	11	10.2	

LC: linguiform vulvar morph type C; LA: linguiform vulvar morph type A; LB: linguiform vulvar morph type B.

## Data Availability

Nematodes samples and Genomic DNA were stored in Tanzania Parasite Resource Bank and the International Parasite Resource Bank (iPRB) in Korea and obtainable on special request. All sequences from the *Haemonchus contortus* isolates from sheep were deposited in GenBank with accession numbers OK236357, OK178541, OK178542, OK178543, OK236356, and OK236358 for the male, knobbed morph type, linguiform A, linguiform B, smooth morph type, and linguiform C, respectively. In addition, the accession numbers OK181226, OK181227, and OK181228 for knobbed, linguiform A, and smooth morph type, respectively, can be retrieved.
